# The effect of relationship quality on individual perceptions of social responsibility in the US

**DOI:** 10.3389/fpsyg.2015.00781

**Published:** 2015-06-10

**Authors:** Joseph C. Thornton

**Affiliations:** Rubel School of Business, Bellarmine University, Louisville, KYUSA

**Keywords:** social responsibility, relationship quality, general self-efficacy, conscientiousness, individual perceptions

## Abstract

Social responsibility (SR) has been of continuing interest in the U.S. and around the world. Organizations make a wide variety of SR decisions that represent differing viewpoints. While a number of definitions of SR exist, many of these definitions indicate that SR decisions may be viewed as existing of various facets, such as legal/regulatory, financial/economic, ethical, environmental, and voluntary. While drivers of SR have been proposed, there has been limited research at a micro-level on how individuals perceive SR activities by the organizations where they work. Based on a prior qualitative study (Thornton and Byrd, 2013) that found SR decisions are related to several traits and influenced by relationships, a model was proposed and tested in this research. The traits found relevant in the qualitative research were conscientiousness, especially in the sense of being responsible, and self-efficacy. Relationship quality was assessed based on positive and negative emotional attractors as proposed in intentional change theory. Perceptions of individuals in management and non-management showed that relationship quality mediated the effect of conscientiousness and general self-efficacy on the SR. Because there are multiple facets, the author made use of Carroll’s (1991) pyramid of SR to identify activities that business owners and managers consider relevant. The findings indicate that conscientiousness is related to specific SR activities in the areas of legal/regulatory, ethical and discretionary dimensions while general self-efficacy is related to financial/economic and legal/regulatory dimensions. The presence of relationship quality enhanced the effects of both conscientiousness and general self-efficacy on the various SR dimensions. This suggests that individuals perceived SR activities along different traits and that enhancing these traits might improve perceptions of SR decisions.

## Introduction

Social responsibility (SR) has been the subject of numerous studies (Bowen, 1953; Aguinis and Glavas, 2012). Most research has focused on large companies and organizations (Williamson et al., 2006) while research into smaller companies has lagged, especially in the U.S. (Dean et al., 1998). European literature has focused on small and medium enterprises (SMEs) in terms of management (Jenkins, 2006), resources (Aragón-Correa et al., 2008), and drivers and determinants (Darnall et al., 2009). A summary of these dissimilarities are as follows.

Fassin (2008) suggests that small firms do not have the resources or capabilities of performing SR activities at the same level as large firms. While Jenkins (2006) indicates that there is more dependence on the values and traits of the owners and senior managers in terms of SR resulting in different responses compared to large firms. A leading driver for small firms is their employees and families as opposed to external stakeholders (Murillo and Lozano, 2006; Darnall et al., 2009).

While there are many definitions for SR (Dahlsrud, 2008), the author uses a broad definition proposed by Bowen (1953, p. 6) that businesses have an obligation to society to “…pursue those policies, to make those decisions, or to follow those lines of action which are desirable in terms of the objectives and values of our society.” The major issue with a broad definition is in determining what an organization might consider the most appropriate policies or objectives that should be addressed. The concept of saliency, developed by Agle et al. (1999) is an attempt to provide a way for organizations to determine which issues should be addressed. They propose that the owners/managers evaluate each issue based on their perception of power, legitimacy, and urgency of specific stakeholders. Based on this, power is seen as the influence of the stakeholder on the organization, legitimacy is based on the perceived relationship quality and urgency is the perception of how the stakeholder sees the issue.

Thornton and Byrd (2013) developed a social empathy model of SR, finding four distinct dimensions of SR that SME owners and managers found important. The dimensions correspond to financial/economic, legal/regulatory, ethical, and discretionary. Internally, the focus is on relationships with employees, suppliers and customers. Externally, the focus is on the community and local/regional issues. This study focuses on the question of how individuals perceive SR actions in the presence of relationship quality. This research also examines whether conscientiousness and general self-efficacy affect SR equally or differentially.

### Conceptual Framework

According to the theory of reasoned action, individuals make decisions based on two major factors, the beliefs and attitudes about the behavior and subjective norms (Ajzen and Fishbein, 1980, 2005). Influences of personal beliefs and attitudes of people relate to their personality, their perception of the various alternatives available, and anticipation of the outcome of the action/decision. Subjective norms reflect the opinions of significant others about the action/decision. It includes the desire for organizational members to “fit in” and comply with these norms. Individuals make decisions from among a limited number of alternatives that they know, learn or experience through interactions with others. In terms of beliefs, managers and owners will anticipate congruence of consequences of actions/decisions with these shared norms and beliefs. Both internal and external norms affect these forces (Ajzen and Fishbein, 1980, 2005). While these occur within individuals, the consequences can be for the entire organization and its strategy (Conley and Williams, 2005; Cordano et al., 2009).

In evaluating SR decisions, Campbell (2007) claims that one reason may be to exploit tax or other group incentives. These in turn may aid the organization or justify its entry into specific professional societies. Others argue that involvement in SR may be a way to strategically position the firm (Keim, 1978; Besser and Miller, 2004). Others claim that it has motivational and marketing value in that it attracts employees (Rupp et al., 2006; Rodrigo and Arenas, 2008) or helps the organization in its image in the community (Bowen, 2000, 2002).

Using a grounded theory approach, Thornton and Byrd (2013) found that owners and managers of SMEs used social and personal values as well as experience in making decisions about SR. They found relationships often triggered compassionate and visionary SR responses from the organization toward individuals or groups both internally or externally. The study also found that owners and managers who were aware of key stakeholders and confident were more likely to enact SR. This is supported by prior research into personal values (Nonis and Swift, 2001), management attitudes (Marshall et al., 2005), and personality (Hogan et al., 1996; Moberg, 1999; Giberson et al., 2005) and their effect on SR decisions.

### Social Responsibility

According to Carroll (1991), every business performs SR activities that are economic and compliance oriented since these are required to remain viable. Carroll (1991) argues that ethical and philanthropic SR dimensions are voluntary in nature. There is some disagreement with Carroll’s original concept of four overlapping dimensions (Schwartz and Carroll, 2003) since the original publication, including that they are poorly defined and that philanthropic responsibilities are potentially not a true form of SR. Schwartz and Carroll (2003) note that the four factor model “remain (s) a leading paradigm of CSR in the social issues in management field.” Dahlsrud in an evaluation of 37 definitions of SR found five conceptual dimensions that occurred with frequencies greater than 50% (stakeholder, social, economic, voluntariness, and environmental dimensions). This lends support to a multidimensional conceptualization of SR.

Thornton and Byrd (2013) found, in interviews with business owners and managers that they want to do the right thing for their employees, customers, and society at large. In particular, many organizations want to make a difference or create an impact on society. Interviews with managers and owners conducted by Thornton and Byrd (2013) found that ethical treatment of employees/customers, recycling/cost reduction, legal compliance, and volunteering/philanthropy were the most frequently discussed decisions for SME engagement. These decisions were mapped to Carroll’s four dimensions of financial/economic (recycling/cost reduction), legal/regulatory (compliance), ethical (employee/customer treatment), and discretionary (volunteering/philanthropic).

### Relationship Quality and SR

For SR decisions and actions to occur, there have to be norms and values supporting and even encouraging them. The norms are expressed through how people act with each other and the nature of their relationships. Boyatzis (2008) and Schwartz and Carroll (2003) describe how sustained, desired change emerges through a complex system. The emergence of each stage in the process in organizations appears to be invoked by a tipping point in the mood of the people in the organization that Boyatzis (2013) referred to as shifts between a negative and a positive emotional attractor (see other papers in this special topic for more detailed explanation of these states and their dynamics). Thornton and Byrd (2013) found in interviews that owners and managers in about 1/3 of the cases indicated that they began or increased SR after a negative emotional event focused them on a particular issue.

While the ultimate decision may rest with upper management, people in an organization would have to value and perceive the desirability and justification of the decisions and actions in a similar manner. This is where the nature and quality of their relationships become the enabling factors. Each person is pulled into a mood state by the degree to which they believe their relationships as having shared compassion (SC), shared vision (SV), and a shared overall positive mood (OPM). These are the norms in the relationships that may enhance the likelihood of seeing the desirability for SR or not, and possibly which dimension SR is more important to them. The perception that relationships in the organization are shared means that individuals perceive that the image of the ideal or desired future of the organization is common (Boyatzis, 2013). This shared relationship means that a degree of trust and caring for each other in the organization and that a common or shared view of the future is hopeful and bright.

If there is a sense of shared purpose or vision, people feel a common context and direction. They also experience a positive emotional attractor (PEA) mood state, which is both psychological and physiological (Schwartz and Carroll, 2003). It is the this mood state, characterized by positive affect, increasing intensity of it and neurological activation of the networks, that enables a person to be more open to new ideas, people and moral concerns (Boyatzis et al., 2014). Openness to new ideas and moral concerns can invite SR thoughts and values. The author proposes that relationship quality creates an opening for broader thinking about the organization’s purpose and role in society and the community, resulting in expansion of the mental models of key stakeholders to be broader than investors do.

Relationship quality has been linked to succession in family businesses (Overbeke, 2010) as well as longer term financial success of family businesses (Neff, 2011). Clayton (2009) reported perceived SV one of the two most significant predictors of championing behavior in mergers and acquisitions, and SV was the strongest predictor of the other mediator, autonomous motivation. A patient’s perception of the quality of their relationship to the physician was shown to mediate treatment adherence for Type II Diabetics (Khawaja, 2012). Pittenger et al. (2012) found that the quality of the relationship between managers and information technology teams enhanced the perception of organizational engagement. In addition, Eisenberg and Miller (1987) argue that when positive emotions are stronger than negative emotions, there is an increase in pro-social behavior. According to Brief and Motowidlo (1986), positive affect has been repeatedly shown to have a positive effect on pro-social behavior, such as helping others. They also note that negative affect does not always lead to decreased pro-social behavior.

The nature of the relationships among those in an organization communicates emotions as well as norms and values. Positive relationships appear to invoke a more open rapport that considers not only the needs and interests of others but the broader community due to the nature of the neural and hormonal arousal (Boyatzis et al., 2014). In this manner, SV, SC, and shared OPM might enhance SR decisions and actions. This has been related to more individualistic ways of thinking about the world (Boyatzis et al., 2000).

### Individual Traits

Thornton and Byrd (2013) found that individual CEOs/Owners of SMEs are influenced by their own prior success in attempting new and different things and that prior success led to a sense of self-efficacy in many arenas, but in particular when addressing SR. According to Bandura (1982, 1991, 1998), self-efficacy is the sense that a person believes in their own ability to perform an activity successfully and suggests that self-efficacy would predict future activities within the same general realm, such as SR. Penner et al. (1995) found that self-efficacy played a strong role in why individuals act in a pro-social manner. They suggest that this may be due to people believing that their actions are effective in helping others. They also believe that the consequences of their decisions and actions are their own responsibility. The author posits that self-efficacy is strengthened by relationship quality since relationships are often seen as providing legitimacy to SR related issues and activities. Therefore, the following hypotheses were developed for this study:

H1a: Relationship quality will mediate the positive relationship between self-efficacy and SR.

H1b: Relationship quality will mediate the positive relationship between self-efficacy and legal SR.

H1c: Relationship quality will mediate the positive relationship between self-efficacy and ethical SR.

H1d: Relationship quality will mediate the positive relationship between self-efficacy and Philanthropic/Discretionary SR.

According to Roberts et al. (2005) conscientiousness is often viewed as a broad personality domain made up of a variety of somewhat similar concepts that are related including: industriousness, order, self-control, responsibility, traditionalism, and virtue. Conscientiousness is associated with conformity and self-regulation (Peterson and Seligman, 2004) leadership and effectiveness (Barrick et al., 1993) and as an expression of virtue (Aguilera et al., 2007). Conscientiousness has been used as a predictor of organizational citizenship behavior at individual (Organ and Ryan, 1995) and organizational (Taylor et al., 2010) levels. Brief and Motowidlo (1986) note that conscientiousness, in terms of perseverance, diligence and putting forth extra effort, is prosocial organizational behavior. Conscientiousness is also seen as conforming to values of the organization (Podsakoff et al., 2000) which can be thought of as individual initiative. Thornton and Byrd (2013) found evidence that CEOs/managers were concerned with doing the right thing and making responsible decisions. CEOs/managers worked to promote a sense of responsibility in their employees and an understanding of why it is important to give back. Based on the results of Thornton and Byrd (2013) that people want to do the right thing (be virtuous) be responsible for their actions and persevere, the author hypothesizes that individuals will perceive economic, legal, ethical, and philanthropic/discretionary SR as being related to conscientiousness and that this relationship will be strengthened by relationship quality. Therefore, the following hypotheses were developed for this study:

H2a: Relationship quality will mediate the positive relationship between conscientiousness and SR.

H2b: Relationship quality will mediate the positive relationship between conscientiousness and legal SR.

H2c: Relationship quality will mediate the positive relationship between conscientiousness and ethical SR.

H2d: Relationship quality will mediate the positive relationship between conscientiousness and Philanthropic/Discretionary SR.

## Materials and Methods

The use of self-report data is recommended by Abbott and Monsen (1979) as having a significant advantage in obtaining data for SR. Podsakoff and Organ (1986) noted that while self-reports may be considered soft data, they are useful for obtaining data related to past behaviors, personality traits, perceptions, and demographics, although researchers should be aware of potential issues present in self-report data. Podsakoff and Organ (1986) noted that one way of increasing reliability is to make the responses anonymous in nature. Therefore, a self-administered on-line survey was used to obtain perceived behaviors, personality traits and demographics of individuals. The survey was designed to avoid collecting any information that might be used to identify specific organizations or individuals resulting in a significant amount of anonymity for respondents. The study was IRB exempt at the University where I was doing my doctoral program, but all human subjects ethical protocols were followed.

### Measures

#### Relationship Quality

To assess the quality of the relationships the positive and negative emotional attractors (PNEA) survey developed by Boyatzis and Oliver (2008) was used to measure the three dimensions of interest: perceived SV, perceived SC, and perceived shared positive mood (PM). The PNEA scale consists of 20 items measured using a 5-point Likert scale: SV (eight-items), SC (six items), and OPM (six items) and has been shown to have good psychometric properties with Cronbach alphas of 0.94, 0.83, and 0.91, respectively, (Pittenger et al., 2012).

#### General Self-Efficacy

The general self-efficacy scale used was developed by Chen et al. (2001). It consists of eight items scored using a 5-point Likert scale. This scale is unidimensional according to Chen et al. (2001) and exhibits good internal consistency and reliability (α = 0.86–0.90) in prior work.

#### Conscientiousness

The conscientiousness scale used was obtained from the international personality item pool (Goldberg et al., 2006), based on the work of Saucier (1997). This scale, consists of ten items (five items reverse scored), was evaluated using a 5-point Likert scale. It was found to have good reliability and internal consistency (α = 0.75) according to Saucier (1997).

#### Social Responsibility

Social responsibility behavior was assessed through two separate scales. One scale developed by Maignan and Ferrell (2000) based on Carroll’s (1991) four dimensions of SR consists of four scales: economic, legal, ethical, and philanthropic. The other scale was developed by Goll and Rasheed (2004) measures discretionary SR. Each scale used a 5-point Likert scale. According to Maignan and Ferrell (2000), the composite reliability (CR) of the overall four dimensional construct was greater than 0.85 and the Cronbach alpha was 0.94. The discretionary SR scale had good psychometrics, with an internal consistency of 0.74 in the original sample (Goll and Rasheed, 2004).

Potential control variables selected based on a review of literature included: job tenure, company tenure, and individual age. Demographic information collected included: current job (management/non-management), company ownership (public/private), and gender. Respondents were asked to identify if the company was a U.S. or foreign company, and to select their industry (food or beverage).

### Sample

The target population for this study includes both publicly and privately owned organizations in the US food and beverage industry. This industry was selected based on the industry focus on customer service and product quality. The data collection was conducted on-line using Qualtrics, Inc. software. The initial data were collected from 308 people. One hundred and ten cases were dropped because the surveys were less than 50% complete. Twenty cases were from outside the US and dropped for consistency of the sample and to control for culture. Eleven cases had more than 500 employees and were dropped to control for size of organization. Ten cases were missing demographic information, and eight cases were from non-food or beverage industries.

The final sample consisted of 149 respondents, with a mean age of 40.4 years (SD 14.3 years), 84 were female, 81 were in management positions, representing 11 public companies, and 138 private companies (including non-profits).

### Preparatory Data Analysis

The presence of multivariate non-normality was excessive for use with covariance-based structural equation modeling (SEM), exceeding the level suggested by Byrne (2010) of less than 7. Thus, further analysis of the data was conducted using partial least squares structural equation modeling (PLS-SEM), which is non-parametric and robust (Ringle et al., 2005; Hair et al., 2010).

A confirmatory factor analysis (CFA) of the data was completed in SmartPLS. When used for CFA, PLS provides evidence of convergent and discriminant validity of the measurement model. In particular, the program provides factor loadings for each measurement and *t*-statistics for the significance of the loading to the latent variables. Measurement variables that had loadings that were not significant were dropped from the analysis.

The final measurement model showed good discriminant validity with no evidence of significant cross loading by measures on other factors. Convergent validity is demonstrated by average variance extracted (AVE) values above 0.50 and CR values that are greater than AVE values, while discriminant validity is demonstrated by loadings of individual variables only on the appropriate latent variables. **Table 1** provides the construct correlations, means, SD, and Cronbach alphas. While there is always some bias related to surveys, allowing individuals a choice of completing a survey results in fewer external validity threats.

**Table 1:** Latent factor correlations (*n* = 149, Cronbach’s α on the diagonal).

Variable	Mean	SD	1	2	3	4	5	6	7
Relationship quality	0.035	0.98	**0.76**						
General self-efficacy	4.11	0.82	0.43	**0.89**					
Conscientiousness	3.76	0.92	0.29	0.08	**0.80**				
Discretionary social responsibility (SR)	3.53	1.05	0.43	0.17	0.47	**0.89**			
Ethical SR	3.73	1.03	0.59	0.14	0.41	0.62	**0.83**		
Legal SR	4.00	0.89	0.56	0.31	0.32	0.53	0.55	**0.80**	
Economic SR	3.62	0.98	0.41	0.34	0.34	0.58	0.52	0.58	**0.71**

### Common Method Bias/Variance

Because the data were gathered using a single instrument, the presence of common method variance (CMV) may be a potential bias. CMV was assessed using the Lindell and Whitney (2001) marker variable technique. CMV was non-significant at a 0.05 level of significance.

### Structural Analysis

The data from this study were analyzed using SmartPLS v2.0 M3 (beta; Ringle et al., 2005). The PLS method provides standardized betas and *R*^2^ values relative to the outer and inner models, where the outer model represents the measurement model and the inner model represents the structural model. Bootstrapping determined the level of statistical significance (*t*-statistics), AVE, CR, Cronbach alpha (α), communality, and redundancy in the model. The reliability, validity, betas, and *R*^2^ values indicate good model fit. The fit assessment was performed using a blindfolding technique as discussed in Tenenhaus et al. (2005). According to Wold (1982), the model is run while a selected construct is removed at specified intervals ranging between 5 and 10, where the interval is not divisible into the sample size. An omission factor of seven was selected for this evaluation. This allowed the creation of cross-validated communalities (*Q*^2^) for each latent variable, where the *Q*^2^ values (greater than zero) indicated that the latent variables were well constructed and results were relevant and predictive (Tenenhaus et al., 2005).

## Results

**FIGURE 1:**
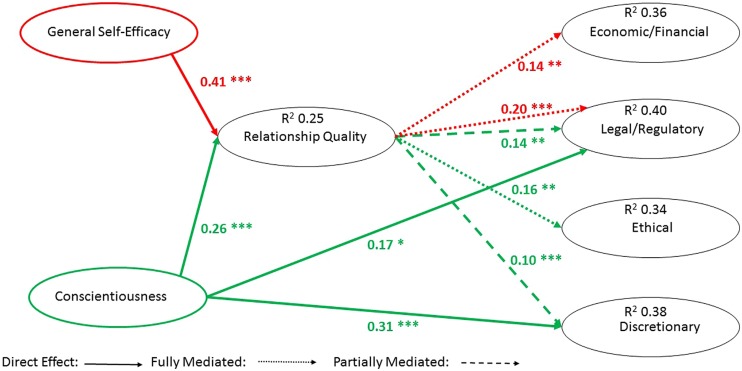
**Results of structural equation modeling (SEM)**. *p < 0.05, **p < 0.01, and ***p < 0.001.

The results of the analysis shown in **Figure 1**, include standardized betas, *p*-values, and type of mediation present.

**Figure 1** shows direct and mediated paths that are significant based on the hypotheses tested, with the presence of the mediating variable. **Table 2** provides the results of the hypothesis testing showing hypotheses that are supported, if the support was direct or mediated, and the type of mediation.

**Table 2:** Summary of hypotheses tested.

Hypothesis	Direction	Support	Std. β (α)	Mediation	Sobel (α)
**H1a:** *Relationship quality will mediate the positive relationship between general self-efficacy and economic SR.*	+	Yes	0.14 (0.003)	Full	2.70 (0.003)
**H1b:** *Relationship quality will mediate the positive relationship between general self-efficacy and legal SR.*	+	Yes	0.20 (<0.001)	Full	3.51 (<0.001)
**H1c:** *Relationship quality will mediate the positive relationship between general self-efficacy and ethical SR.*	+	No	Not significant		
**H1d:** *Relationship quality will mediate the positive relationship between general self-efficacy and discretionary SR.*	+	No	Not significant		
**H2a:** *Relationship quality will mediate the positive relationship between Conscientiousness and economic SR.*	+	No	Not significant		
**H2b:** *Relationship quality will mediate the positive relationship between Conscientiousness and legal SR.*	+	Yes	0.14 (0.002) 0.17 (0.038)	Partial Direct	2.80 (0.002)
**H2c:** *Relationship quality will mediate the positive relationship between Conscientiousness and ethical SR.*	+	Yes	0.16 (0.002)	Full	2.93 (0.002)
**H2d:** *Relationship quality will mediate the positive relationship between Conscientiousness and discretionary SR.*	+	Yes	0.10 (0.003) 0.31 (<0.001)	Partial Direct	2.74 (0.003)

Using Cohen’s f^2^ (Soper, 2012), the effect size of the regression coefficients was determined to be moderate for relationship quality (0.33), and large for economic (0.57), legal (0.52), ethical (0.61), and discretionary (0.66) SR. The data indicate that a significant positive correlation exists between conscientiousness, general self-efficacy, and the various SR dimensions and that Relationship quality strengthens this effect. The mediation effect was evaluated following the method of Baron and Kenny and the effects were assessed using the Sobel test (Baron and Kenny, 1986; Hayes, 2009; Soper, 2010).

## Discussion

The purpose of this study was to assess selected individual characteristics and relationship quality in their effect on individual perceptions of SR decisions along four conceptual dimensions. The major contribution of the study suggests that efforts at stimulating increases in SR might focus on fostering self-efficacy and conscientiousness along with creating higher quality relationships in terms of SV, compassion and PM within organizations as opposed to a broad appeal to social conscience. The findings indicate significant positive direct relationships of self-efficacy and conscientiousness with different SR dimensions. These connections are strengthened through the relationship that individuals perceive to exist within their organization.

This study found that conscientiousness, manifested as being responsible, is positively related to legal and discretionary SR both directly and when partially mediated by relationship quality. Conscientiousness is related to ethical SR and fully mediated by relationship quality. The link between conscientiousness and economic SR is not significant. This could be due to the essential pragmatic nature that would drive economic SR with its enhanced beliefs in individual effort. This tendency to see things as individualistic may be at odds with a more socially concerned sense of duty emerging from conscientiousness.

Self-efficacy is positively related to economic and legal SR and fully mediated by relationship quality. Regarding economic SR, this complements the non-significant findings for conscientiousness explained above. The stronger relationship of self-efficacy to economic and legal SR does seem to be a function of more individualistic and utilitarian nature of economic perspectives (Boyatzis et al., 2000). Legal SR is often a more task-oriented perspective, rather than the more philosophical and larger scale perspectives involved in ethical and discretionary SR.

These findings suggest that regardless of individual dispositions people perceive SR, the quality of one’s relationships enable multiple aspects of SR. Further research may show how SR can be motivated through emotional and social contagion. The full mediation of ethical SR is indicative of emotional involvement through SV pulling the individuals toward a focus on future opportunities (Goleman et al., 2001; Goleman, 2006). In such settings, relationships with a SV and compassion arouse intrinsic motivation. Howard (2006) noted that the creation of positive emotions serves to pull a person toward the ideal self or their personal vision, shaping the response toward goals and behaviors that correspond to our intrinsic values and behaviors. This deflates, somewhat, the argument that ethical and discretionary SR is only a form of marketing or image self-interest by the organization.

Based on the positive aspects of SV, compassion, and PM, it would appear that various dimensions of SR might be experienced as intrinsic motivators of the SR of organizations. Dutton et al. (2007) argue that compassion is particularly important in organizations, since it increases interconnections between employees leading to greater levels of trust and enthusiasm to acting in a positive manner. Relationship quality serves to create a linkage between individuals and the organizations resulting in the organization noticing issues, feeling with others and finally taking action on those issues.

### Limitations

One limitation of this study is the size and nature of the population surveyed. The relationships to different dimensions of SR discovered may be different in large companies, public sector organizations or non-profits. The use of a single type of data (self-report) is known to create potential for CMV, which was addressed in this study using a marker variable technique (Lindell and Whitney, 2001).

### Implications for Practice and Research

Further research is needed to test the linkage between individuals and organizational SR along specific dimensions, especially in terms of the effect of positive and negative emotions. In addition, subsequent research should investigate other personality dispositions, like agreeableness, to determine if they have an impact of different facets of SR, or even traits like general mental ability. Given the results of this study, a follow-up study should examine the differential effects of SV, SC, and shared PM. It would also be desirable to study composite views of people in an organization on their perceptions of each SR dimension and relate this to individual traits and perceptions.

## Conclusion

The appeal to stimulate more SR along each of the four dimensions in organizations would be enhanced if people worked on the degree of SV and compassion in their relationships. Whether a person acts with SR is often attributed to some individual characteristic, trait, or value. This study examined how the nature of relationships may alter perceptions of corporate responses leading to different dimensions of SR activities. In this sense, the development of better relationships in terms of SV, compassion, and PM may help promote various forms of SR. As discussed by (Kanov et al., 2004), the sense of SC serves to link the individual responses within the organization to the overall response of the organization to the feelings and needs of others. This has a strong effect on the sense of responsibility (conscientiousness) that people have when they make decisions and may lead to more caring organizations.

## Conflict of Interest Statement

The author declares that the research was conducted in the absence of any commercial or financial relationships that could be construed as a potential conflict of interest.
